# DHCR24 INSUFFICIENCY PROMOTES VASCULAR ENDOTHELIAL CELL SENESCENCE AND ENDOTHELIAL DYSFUNCTION

**DOI:** 10.1093/geroni/igae098.2440

**Published:** 2024-12-31

**Authors:** Han Li, Zhen Yang, Wukaiyang Liang, Hao Nie, Jinhua Yan, Cuntai Zhang

**Affiliations:** Key Laboratory of Vascular Aging, Ministry of Education,Tongji Hospital of Tongji Medical College, Huazhong University of Science and Technology, Wuhan, Hubei, China (People’s Republic); Key Laboratory of Vascular Aging, Ministry of Education,Tongji Hospital of Tongji Medical College, Huazhong University of Science and Technology, Wuhan, Hubei, China (People’s Republic); Key Laboratory of Vascular Aging, Ministry of Education,Tongji Hospital of Tongji Medical College, Huazhong University of Science and Technology, Wuhan, Hubei, China (People’s Republic); Key Laboratory of Vascular Aging, Ministry of Education,Tongji Hospital of Tongji Medical College, Huazhong University of Science and Technology, Wuhan, Hubei, China (People’s Republic); Key Laboratory of Vascular Aging, Ministry of Education,Tongji Hospital of Tongji Medical College, Huazhong University of Science and Technology, Wuhan, Hubei, China (People’s Republic); Tongji Hospital, Tongji Medical College, Huazhong University of Science and Technology, Wuhan, Hubei, China (People’s Republic)

## Abstract

Endothelial cells (ECs) senescence is critical for vascular dysfunction, which leads to age-related disease. DHCR24, a 3β-hydroxysterol δ 24 reductase with multiple functions other than enzymatic activity, has been involved in age-related disease. However, little is known about the relationship between DHCR24 and vascular ECs senescence. We revealed that DHCR24 expression is chronologically decreased in senescent human umbilical vein endothelial cells (HUVECs) and the aortas of aged mice. ECs senescence in endothelium-specific DHCR24 knockout mice was characterized by increased P16 and senescence-associated secretory phenotype, decreased SIRT1 and cell proliferation, impaired endothelium-dependent relaxation, and elevated blood pressure. In vitro, DHCR24 knockdown in young HUVECs resulted in a similar senescence phenotype. DHCR24 deficiency impaired endothelial migration and tube formation and reduced nitric oxide (NO) levels. DHCR24 suppression also inhibited the caveolin-1/ERK signaling, probably responsible for increased reactive oxygen species production and decreased eNOS/NO. Conversely, DHCR24 overexpression enhanced this signaling pathway, blunted the senescence phenotype, and improved cellular function in senescent cells, effectively blocked by the ERK inhibitor U0126. Moreover, desmosterol accumulation induced by DHCR24 deficiency promoted HUVECs senescence and inhibited caveolin-1/ERK signaling. Our findings demonstrate that DHCR24 is essential in ECs senescence.

